# Distinct Community Composition of Previously Uncharacterized Denitrifying Bacteria and Fungi across Different Land-Use Types

**DOI:** 10.1264/jsme2.ME19064

**Published:** 2020-01-30

**Authors:** Reiko Fujimura, Yoichi Azegami, Wei Wei, Hiroko Kakuta, Yutaka Shiratori, Nobuhito Ohte, Keishi Senoo, Shigeto Otsuka, Kazuo Isobe

**Affiliations:** 1 Graduate School of Agricultural and Life Sciences, The University of Tokyo, Tokyo 113–8657, Japan; 2 Jiangsu University, Jiangsu 212013, China; 3 Niigata Agricultural Research Institute, Niigata 940–0826, Japan; 4 Graduate School of Informatics, Kyoto University, Kyoto 606–8501, Japan; 5 Collaborative Research Institute for Innovative Microbiology, The University of Tokyo, Tokyo 113–8657, Japan

**Keywords:** denitrification, nitrite reductase gene, *nirK*, *nirS*

## Abstract

Recent studies demonstrated that phylogenetically more diverse and abundant bacteria and fungi than previously considered are responsible for denitrification in terrestrial environments. We herein examined the effects of land-use types on the community composition of those denitrifying microbes based on their nitrite reductase gene (*nirK* and *nirS*) sequences. These genes can be phylogenetically grouped into several clusters. We used cluster-specific PCR primers to amplify *nirK* and *nirS* belonging to each cluster because the most widely used primers only amplify genes belonging to a single cluster. We found that the dominant taxa as well as overall community composition of denitrifying bacteria and fungi, regardless of the cluster they belonged to, differed according to the land-use type. We also identified distinguishing taxa based on individual land-use types, the distribution of which has not previously been characterized, such as denitrifying bacteria or fungi dominant in forest soils, *Rhodanobacter* having *nirK*, *Penicillium* having *nirK*, and *Bradyrhizobium* having *nirS*. These results suggest that land-use management affects the ecological constraints and consequences of denitrification in terrestrial environments through the assembly of distinct communities of denitrifiers.

Denitrification is performed by diverse microbes. These microbes utilize soluble nitrogen (N) oxides (NO_3_^–^ and NO_2_^–^) and produce gaseous N compounds (NO, N_2_O, and N_2_) in a stepwise process: NO_3_^–^ → NO_2_^–^ → NO → N_2_O → N_2_. Denitrification may widely occur in terrestrials and has attracted attention because of its environmental impact. A large portion of nitrogen in the nitrogenous fertilizers applied to agricultural land may be lost through denitrification (Mulvaney *et al.*, 1997). N_2_O is a potent greenhouse gas and an ozone-depleting substance (Braker *et al.*, 1998; Ravishankara *et al.*, 2009).

*nirK* and *nirS* are frequently used as markers for the detection and phylogenetic identification of denitrifying microbes. Denitrifiers contain one of two functionally equivalent, but structurally different, oxidoreductases that catalyze the reduction of NO_2_^–^ to NO, *i.e.*, the copper-containing reductase (NirK) or cytochrome cd1-containing reductase (NirS), with some exceptions that may possess both enzymes (Rinaldo and Cutruzzolà, 2007; Kits *et al.*, 2015). Denitrifiers can be phylogenetically grouped based on their *nirK* and *nirS *sequences into several clusters, as shown in Fig. 1A and C.

We recently developed cluster-specific PCR primers to amplify *nirK* and *nirS* belonging to each cluster because the most widely used primers (hereafter referred to as conventional primers), designed approximately two decades ago (Braker *et al.*, 1998; Hallin and Lindgren, 1999), only amplify *nirK* in Cluster I and *nirS* in Cluster I, as shown in Fig. 1A and C (Wei *et al.*, 2015a). We subsequently reported that *nirK* and *nirS* in terrestrial environments were more phylogenetically diverse and 2-6-fold more abundant than those revealed with conventional primers (Wei *et al.*, 2015a). Furthermore, we found that bacterial denitrifiers with *nirK* in Clusters I and II and *nirS* in Cluster I as well as fungal denitrifiers with *nirK* in Cluster V were responsible for N_2_O emission in fertilized cropland soils (Wei *et al.*, 2014; 2015a; 2015b). DNA stable-isotope probing coupled with metagenomic shotgun sequencing also revealed that bacterial denitrifiers with *nirK* in Cluster II were actively involved in denitrification in agricultural soils (Coyotzi *et al.*, 2017).

The geographic distribution of denitrifiers has been investigated in an attempt to gain insights into the microbial control of denitrification (Enwall *et al.*, 2010) because the spatial distribution of denitrifiers may represent the denitrification rate in different environments (Philippot *et al.*, 2009). However, it currently remains unclear whether denitrifiers that can be detected using cluster-specific PCR primers show biogeographic patterns that are consistent with those identified using conventional primers. In the present study, we assessed the geographic distribution of denitrifiers based on their *nirK* and *nirS* sequences and assigned taxonomies in various terrestrial environments. We specifically investigated whether the land-use type affects the composition of abundant denitrifiers. We previously performed a small-scale survey, in limited locations, of bacterial *nirK* and *nirS* using a clone library analysis (Wei *et al.*, 2015a), which generated insufficient data to answer this question. Therefore, in the next stage, which we report here, we performed the high-throughput sequencing of genes from a wider variety of locations. We focused on denitrifiers with *nirK* in Clusters I, II, and V and *nirS* in Cluster I because they were abundant in our pilot survey and are primarily responsible for denitrification in croplands (Wei *et al.*, 2014; 2015a; 2015b).

## Materials and Methods

### Samples

Soil samples were taken from rice paddy fields, croplands, and forests (Table 1). Aeration by water-logging or oxygen transfer in paddy fields and N fertilization in croplands may markedly alter the microbial community composition in soils (Jangid *et al.*, 2008). Furthermore, the microbial community composition of forest soils is geographically highly variable (Fierer and Jackson, 2006). Thus, in order to capture as diverse *nirK* and *nirS* as possible in terrestrials, we used rhizosphere (Paddy 1 and 2) and non-rhizosphere (bulk soil; Paddy 3–6) soils collected during flooding (Paddy 1, 4–6) and intermittent irrigation (Paddy 2 and 3) periods in a paddy field. We also used N-fertilized soils, *i.e.*, one without fertilizer (Cropland 1), one fertilized with urea (Cropland 2), and one fertilized with organic manure (Cropland 3) collected from cropland, and forest soils (Forest 1–6) collected from different locations. Rhizosphere soils were collected from rice plant roots. We vigorously hand-shaked rice roots and collected the remaining soil attached to the roots as rhizosphere soils. The soils in rice paddy fields and croplands were collected at a depth of 5–10 cm below the surface from three randomly selected spots using a core sampler, and were then pooled in a plastic bag. Forest soil was collected from one spot in each location, as described previously by Urakawa *et al.* (2015).

In a subsequent analysis of *nirK* in Cluster II, we also collected non-rhizosphere paddy soils during intermittent irrigation periods (Paddy 5 and 6) and N-fertilized cropland soils (Cropland 4, 5, and 6) from different geographic locations (Table 1) with the same sampling method as that described above. The sampling depth for all soils was 5–10 cm below the surface.

All samples were stored at –20°C until used. Details of sampling locations, soil conditions, soil types, and *nir* clusters analyzed are shown in Table 1.

### DNA extraction, PCR amplification, and high-throughput sequencing

Microbial DNA was extracted from 0.4–0.5 g of samples using an ISOIL for the Beads Beating kit (Nippon Gene), according to the manufacturer’s instructions.

*nirK* and *nirS* were amplified with each primer set for *nirK* in Clusters I (nirKC1F/nirKC1R), II (nirKC2F/nirKC2R), and V (nirKfF/nirKfR) and *nirS* in Cluster I (nirSC1F/nirSC1R) (Wei *et al.*, 2015a; 2015b). PCR reaction mixtures (final volume 50 μL) contained 1×Immo Buffer (Bioline Pty), 4‍ ‍mM MgCl_2_, 0.2‍ ‍mM dNTP mix, 0.2 μM of each primer, 0.5 μg μL^–1^ bovine serum albumin, 0.02 U μL^–1^ BioTaq (Bioline Pty), and 10 ng of environmental DNA. Amplification reactions were performed with a Veriti thermal cycler (Applied Biosystems) under the following conditions: an initial denaturation step at 95°C for 5 min, followed by 30 cycles at 95°C for 30‍ ‍s, 54–60°C (annealing temperature depending on the primer sets; see Wei *et al.* (2015a) and Wei *et al.* (2015b)) for 30‍ ‍s, and 72°C for 30 s. PCR products were purified using a Wizard SV Gel and PCR Clean-Up System (Promega). The sequence libraries for the multiplex sequencing analysis using the Illumina Miseq system (Illumina) were prepared using a NEBNext Ultra DNA Library Prep kit for Illumina with NEBNext Multiplex Oligos for Illumina (New England Biolabs). SPRIselect (Beckman Coulter) was used as a cleanup step for the better selection of fragment sizes. The quantification and quality of libraries were checked using a KAPA Library Quantification Kit (KAPA Biosystems) and an Agilent 2100 Bioanalyzer (Agilent Technologies). Multiplex sequencing runs contained the indexed PCR products of all samples that were combined with equal concentrations. Paired-end sequence analyses of 300 cycles with the Illumina Miseq sequencer were performed according to the manufacturer’s protocol. The total sequence read counts of each sample were shown in Table S1.

### Sequence analysis

The UPARSE pipeline (Edgar, 2013) was used to merge de-multiplexed sequences and conduct quality filtering. We set a minimum overlap of 30 bp for merging paired-end reads and a minimum length of merged reads to 200 bp. A maximum number of expected errors (E_max) of 0.5 was used to quality-filter sequences, and singletons and doubletons were removed to reduce sequencing noise. Sequence data were sorted by each primer set using fqgrep (https://github.com/indraniel/fqgrep), and the primer-annealing regions of the sequences were removed using the FASTX toolkit (http://hannonlab.cshl.edu/fastx_toolkit). Nucleotide sequences were translated to amino acid sequences using EMBOSS (Rice *et al.*, 2000). Further noise (*i.e.*, short reads or potentially non-*nir* amino acid sequences) was removed using a homology search against the curated reference database of *nirK* or *nirS*. We developed a reference database of *nirK* or *nirS* amino acid sequences by curating the *nirK* or *nirS* database downloaded on 8 August 2015 from the FunGene database (minimum amino acid sequence length, 200; minimum score, 150) (Fish *et al.*, 2013). We used the BLASTP function of the BLAST+ tool (Camacho *et al.*, 2009) for the homology search using the following threshold values: E-value <0.001, alignment length >100 bases. This process also enabled the taxonomic identification of *nirK* and *nirS* in our dataset. We defined denitrifiers with different amino acid sequences for *nirK* or *nirS* as belonging to different taxa. The number of sequences of *nirK* or *nirS* from each cluster was rarefied to 1,850 per sample by random sampling using the Phyloseq package (McMurdie and Holmes, 2013) in the R environment (https://www.r-project.org). The total number of taxa of each sample was shown in Table S2.

### Phylogenetic and alpha- and beta-diversity analyses

**Fig. 1.**
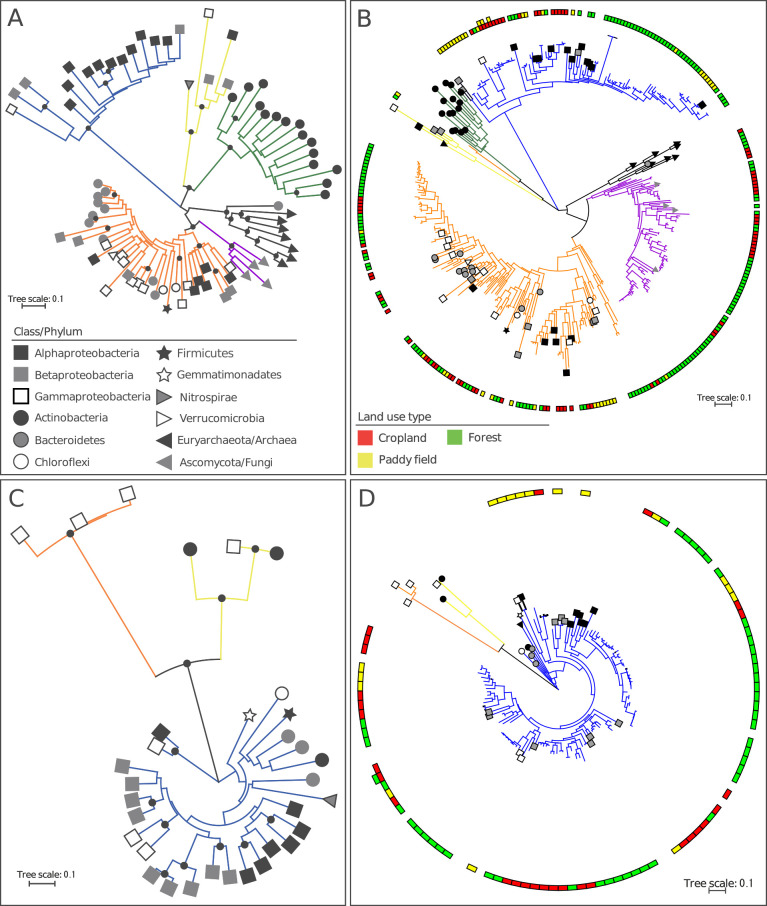
Maximum likelihood phylogenetic trees for reference sequences of *nirK* (A) and *nirS* (C), and environmental amplicon sequences of *nirK* (B) and *nirS* (D), based on their amino acid sequences. Sequences showing >1% relative abundance in each sample were used for B and D. Squares are arranged in a circle on the tree indicating the location of the sequences on the branch node. Legends for the node shapes and colors are shown in the Figures. Branch colors indicate the clusters as follows: blue, Cluster I; orange, Cluster II; green, Cluster III; yellow, Cluster IV; purple, Cluster V; black, halophilic archaea (*nirK* in Cluster II). The solid circles on the branch indicate bootstrap values of >80%.

In the phylogenetic analysis, we initially obtained full-length *nirK* and *nirS* amino acid sequences from the Microbial Genome Database (MBGD) (Uchiyama *et al.*, 2015) and constructed phylogenetic reference trees. These sequences were aligned using Clustal W version 2.0 and maximum likelihood trees were constructed by a bootstrap analysis (500 replicates) using MEGA 5 (Tamura *et al.*, 2011). The reference trees generated 5 and 3 clusters for the *nirK* and *nirS* amino acid sequences, respectively (Fig. 1A and C), as reported previously (Wei *et al.*, 2015a; 2015b). We then extracted abundant amino acid sequences in each cluster that accounted for >1% of the total sequences (>18/1,850 sequences), merged them with the sequences used in the reference trees, and constructed phylogenetic trees in the same manner as the reference trees.

In alpha- and beta-diversity analyses, we used 1,850 sequences per sample. We estimated the alpha-diversities (*i.e.*, the number of taxa and Chao 1 and Shannon–Wiener indexes) of *nirK* (Clusters I, II, and V) and *nirS* (Cluster I) using the “phyloseq” package. We then estimated beta-diversities based on the Bray–Curtis dissimilarity and visualized them with a non-metric multidimensional scaling ordination (NMDS) using the “vegan” package (Oksanen *et al.*, 2017). Since we had additional samples (*i.e.*, Paddy 5–6 and Cropland 4–6 samples) for *nirK* in Cluster II, we reanalyzed beta-diversities using these samples.

### Statistical analysis

Alpha-diversities were compared among the clusters using a one-way analysis of variance (ANOVA) followed by Tukey’s honest significant difference test. Beta-diversities were statistically compared among land-use types by a permutational multivariate analysis of variance (PERMANOVA) test using the “adonis” function in the “vegan” package.

## Results and Discussion

### Phylogenetic distributions and alpha-diversities of *nirK* and *nirS*

*nirK* in Clusters I and II and *nirS* in Cluster I were present by PCR amplification in all land-use types, whereas *nirK* in Cluster V was only amplified from forest and cropland soils. This result revealed the lower abundance of fungal denitrifiers in wetter or more anoxic environments (*i.e.*, paddy soils), which is consistent with previous findings showing that fungi may require oxygen for denitrification and also that fungal denitrification may be more dominant than bacterial denitrification at the surface of cropland soils (Wei *et al.*, 2014). We rarefied the *nirK* and *nirS* sequences to 1,850 reads, and *nirK* in Cluster I from Forest 1 (86 reads) and *nirK* in Cluster V from Forest 6 (763 reads) were then discarded in alpha- and beta-diversity analyses because of the low number of reads (Table S2). The rank abundance curve showed that 1,850 reads covered the dominant taxa in the most diverse *nir* cluster (Fig. S1; Cluster II in *nirK*); therefore, 1,850 reads were sufficient to capture the majority of *nirK* and *nirS* diversities. We obtained non-fungal *nirK* (an average of 48 reads, ranging between 6 and 180, out of 1,850; Table S2) from PCR amplicons using the primers for *nirK* in Cluster V because sequences in the primer annealing region of fungal *nirK* were not completely different from those of bacterial *nirK* (Wei *et al.*, 2015b). The present study focused on the diversity of fungal denitrifiers; therefore, these non-fungal *nirK* sequences were discarded in subsequent analyses.

The phylogenetic distribution of abundant *nirK* and *nirS* (>1% abundance) showed that the amplified sequences were placed in the expected clusters (Fig. 1B and D). Furthermore, the phylogenetic distribution also showed that the *nirK* and *nirS* sequences were unique for each land-use type (Fig. 1B and D), with the three sequences in *nirK* and one in *nirS* overlapping between the different land-use types, suggesting that the phylogenetic distribution of denitrifying bacteria and fungi in the environment differs according to the land-use type.

**Fig. 2.**
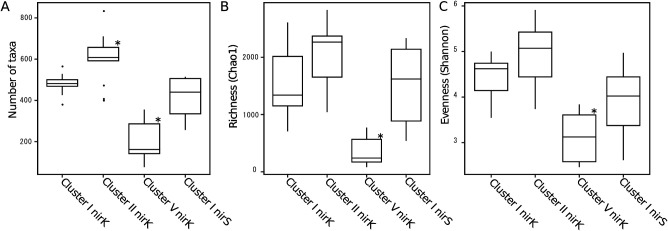
Community diversity indexes: the number of taxa (A), Chao1 richness (B), and Shannon–Wiener diversity index (C), of denitrifying bacteria and fungi having *nirK* or *nirS* belonging to each cluster among all samples. Asterisks denote values that are significantly differently from others (ANOVA; *P*<0.05). The box plot chart shows values within the range between the 1st and 3rd quartiles and the line inside represents the 2nd quartile (median). Whiskers show the lowest and highest values within 1.5 interquartile ranges from the 1st and 3rd quartiles, respectively. Black dots show outliers beyond the whiskers.

The number of taxa, Chao 1 richness, and Shannon index values suggested that *nirK* in Cluster II was the most diverse among the clusters examined (Fig. 2). In contrast, *nirK* in Cluster V was the least diverse among the clusters examined (Fig. 2). The highest diversity of *nirK* in Cluster II clearly showed that cluster-specific primers enabled the detection of more diverse denitrifiers than conventional primers. In addition, no significant differences were observed in alpha-diversities among the soil samples in each cluster examined (data not shown).

### Beta-diversity of denitrifiers having *nirK* and *nirS*

**Fig. 3.**
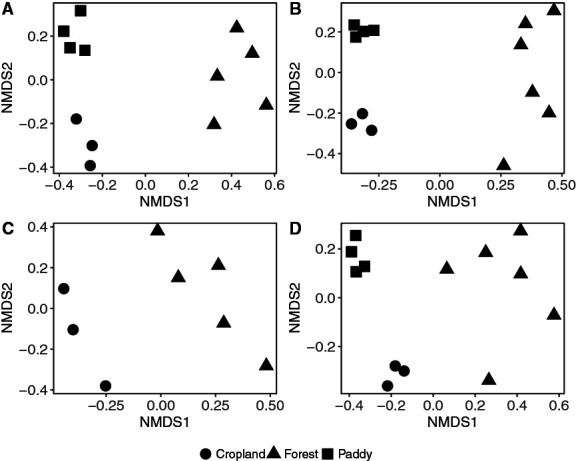
Non-metric multidimensional scaling (NMDS) ordination of variations in communities of denitrifying bacteria and fungi having *nirK* in Cluster I (A), Cluster II (B), and Cluster V (C), and *nirS* in Cluster I (D), based on the Bray–Curtis dissimilarity index among different land-use types. Stress values are 0.07 (A), 0.04 (B), 0.03 (C), and 0.10 (D).

The beta-diversity analysis showed that the composition of denitrifying bacteria and fungi differed according to the land-use type based on *nirK* and *nirS* sequences in all clusters (PERMANOVA: R^2^=0.50, *P*<0.001 for *nirK* in Cluster I; R^2^=0.40, *P*<0.001 for *nirK* in Cluster II; R^2^=0.20, *P*=0.02 for *nirK* in Cluster V; R^2^=0.44, *P*<0.001 for *nirS* in Cluster I) (Fig. 3). To capture as many diverse genes and taxonomic groups as possible in the environment, we selected various environmental conditions for the analysis, including different water regimes in paddy soils and different nitrogen fertilizers in cropland soils. However, these soil samples originated from a single geographic location (Niigata, Table 1). Therefore, we added paddy field and cropland soil samples from other more remote locations (two and three samples each for paddy and cropland soils, respectively) for the analysis of *nirK* in Cluster II. We focused on *nirK* in Cluster II because it was the most diverse, but also an uncharacterized cluster. The results obtained showed that the composition of denitrifiers that possessed *nirK* in Cluster II differed by land-use type (PERMANOVA: R^2^=0.40, *P*<0.001) (Fig. S2). Previous studies using conventional primers suggested that the composition of denitrifiers having *nirK* in Cluster I and *nirS* in Cluster I was sensitive to soil environmental properties, such as pH, nutrient availability, and Cu content, which may be largely characterized by the land-use type (Philippot *et al.*, 2007; Attard *et al.*, 2010). The present results suggest that the land-use type or soil environmental properties (potentially oxygen and nutrient availabilities) shape the composition of denitrifiers regardless of the cluster they belong to.

### Taxonomic assignment of denitrifiers having *nirK* and *nirS*

**Fig. 4.**
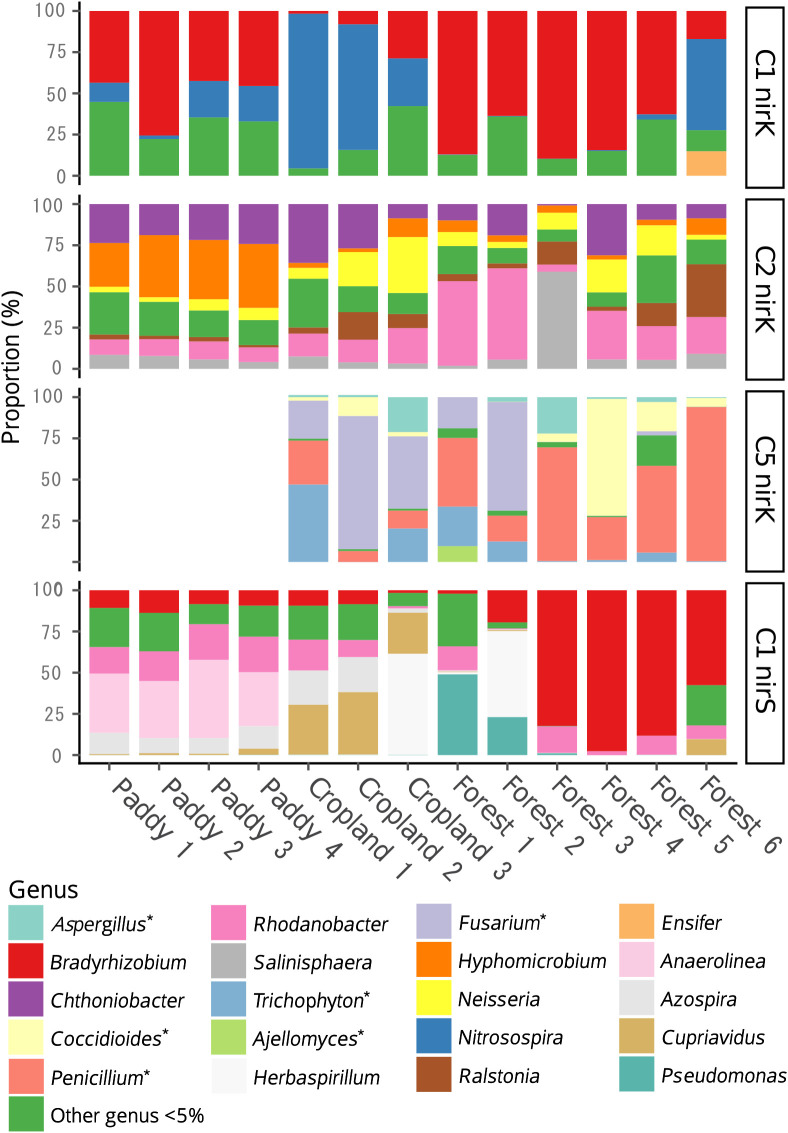
Assigned genus-level taxonomic composition of denitrifying bacteria and fungi having *nirK* or *nirS* belonging to each cluster in each sample. The composition was analyzed based on the rarefied 1,850 reads, except for *nirK* in Cluster I from Forest 6 (86 reads) and *nirK* in Cluster V from Forest 1 (763 reads). The y-axis indicates the relative abundance of the taxa. The color legend is shown in the Figure. Note that C1 *nirK*, C2 *nirK*, C5 *nirK*, and C1 *nirS* represent *nirK* in Cluster I, *nirK* in Cluster II, *nirK* in Cluster V, and *nirS* in Cluster I.

The taxonomic composition of denitrifying bacteria and fungi possessing *nirK* and *nirS* appeared to differ according to the land-use type (Fig. 4). The *nirK* sequences in Cluster I showed the highest similarity mostly with those from the genera *Bradyrhizobium* (the class Alphaproteobacteria) and *Nitrosospira* (the class Betaproteobacteria) (Fig. S3). *Bradyrhizobium*-like *nirK* sequences were dominant in this cluster in forest and paddy soils, whereas *Nitrosospira*-like *nirK* sequences were dominant in cropland soils and were not detected in most forest soil samples.

The *nirK* sequences in Cluster II showed the highest similarity, mostly with those from the genera *Rhodanobacter* (the class Gammaproteobacteria), ‘*Chthoniobacter*’ (the phylum Verrucomicrobia), and *Hyphomicrobium* (the class Alphaproteobacteria) (Fig. S3). *Rhodanobacter*-like *nirK* sequences were dominant in this cluster in most forest soils, whereas *Hyphomicrobium*-like *nirK* sequences were dominant in paddy soils (Fig. 4). ‘*Chthoniobacter*’-like *nirK* sequences were not detected in our previous clone library analysis.

The *nirK* sequences in Cluster V showed the highest similarity mostly with those from the genera *Fusarium*, *Coccidioides*, *Penicillium*, and *Trichophyton* in the class *Ascomycota* (Fig. S3). *Fusarium*-like *nirK* sequences were dominant in this cluster in cropland soils, whereas *Penicillium*-like *nirK* sequences were dominant in forest soils (Fig. 4).

The *nirS* sequences in Cluster I showed the highest similarity mostly with those from the genus *Bradyrhizobium* (the class Alphaproteobacteria), followed by *Rhodanobacter* (the class Gammaproteobacteria), and *Anaerolinea* (the class Chloroflexi) (Fig. S3). *Bradyrhizobium*-like *nirS* sequences were dominant in this cluster in most forest soils, whereas *Anaerolinea*-like *nirS* sequences were dominant in paddy soils (Fig. 4).

The results of the taxonomic composition analysis revealed the distinguishing denitrifying taxa in soils from individual land-use types, the distribution of which has not previously been characterized, such as *Rhodanobacter* having *nirK* in Cluster II, *Penicillium* having *nirK* in Cluster V, and *Bradyrhizobium* having *nirS* in Cluster I. The dominance of *Rhodanobacter*-like *nirK* sequences in forest soils may be attributed to their acidophilic or acid-tolerant ability. Van den Heuvel *et al.* (2010) found that although denitrification was generally inhibited at a pH of less than 5 in pure cultures of denitrifying bacteria, a soil microbial community dominated by *Rhodanobacter* sp. enriched in an acidic reactor performed denitrification, even at pH 4. In addition, the *Rhodanobacter* strains recently isolated from forest soils grew at a wide range of pH (4.5–11.0 for *R. humi* sp. and 4.5–10.0 for *R. hydrolyticus* sp.) (Dahal and Kim, 2017; Dahal *et al.*, 2018). Forest soils are generally acidic (pH ranges were 4.8–6.0, 6.6–6.8, and 5.4–6.6 for forest, paddy, and cropland soils, respectively, in the present study, Table S3), and, thus, soil acidity may enrich denitrifying *Rhodanobacter* sp. in forest soils. The genus *Penicillium*, denitrifying fungi for which *nirK* sequences were dominant in forest soils, may also prefer acidic environments. Maeda *et al.* (2015) cultivated diverse fungal isolates and showed that denitrifying *Penicillium* strains decreased the pH of their growth medium (from pH 7.5 to 3–5) the most among all the strains tested. We also found that *Bradyrhizobium*-like *nirS* sequences (Cluster I) were dominant in most forest soils. *Bradyrhizobium* having *nirS*, and with denitrification capability, was the first to be confirmed among the strains isolated from paddy soil (Ishii *et al.*, 2011). Prior to this, *Bradyrhizobium* strains were known to carry only *nirK* (Ishii *et al.*, 2011). Two *Bradyrhizobium* strains that have both *nirK* and *nirS* were subsequently confirmed, and both were isolated from paddy soils (Jang *et al.*, 2018; Sánchez and Minamisawa, 2018). Therefore, in the present study, forest soils predominantly contained both *Bradyrhizobium*-like *nirS* and *nirK* sequences, whereas paddy soils predominantly contained *Bradyrhizobium*-like *nirK* sequences.

## Conclusion

The present study demonstrated that the land-use type or associated soil environmental properties may shape the community composition of denitrifying bacteria and fungi regardless of the cluster they belong to. The land-use type may also select for distinguishing taxa, possibly based on their physiological adaptations to soil environmental properties.

We used cluster-specific primers because of their high detectability of diverse *nirK* and *nirS* genes. These primers represent the best potential for detecting these genes (Ma *et al.*, 2019), but do not identify genes from archaea (Bonilla-Rosso *et al.*, 2016; Kobayashi *et al.*, 2018) or potentially those from some bacterial or fungal groups (Ma *et al.*, 2019). In addition, the results on taxonomic assignment depend on the fullness of the database. A future increase in database entries, driven by the discovery of novel denitrifiers or denitrifiers having novel *nirK* or *nirS*, such as *Bradyrhizobium* having *nirS*, may modify results. Furthermore, the lateral transfer of genes may make taxonomic assignment challenging (Jones *et al.*, 2008). However, despite these limitations and the need for further improvements, the present results are convincing.

Previous studies suggested that the geographic distribution of denitrifiers reflect the denitrification rate in different environments (Philippot *et al.*, 2009). Future studies are needed to clarify whether and how the microbial denitrifying community control terrestrial denitrification, and how this differs according to the land-use type.

### Nucleotide sequence accession numbers

The nucleotide sequence data reported are available in the DDBJ Sequenced Read Archive under accession numbers DRX115277–DRX115296.

## Supplementary Material

Supplementary Material

## Figures and Tables

**Table 1.** Sampling locations, soil properties, pH, and *nir* clusters analyzed.

Sample name	Location in Japan	Habitat	Soil type	Management	*nir* clusters analyzed	Ref.
Paddy 1	Niigata (37°26'N, 138°52'E)	Bulk soil	Gray lowland	Before irrigation	*nirK* in Clusters I, II, V *nirS* in Cluster I	(Itoh *et al.*, 2013)
Paddy 2		Bulk soil	Gray lowland	After irrigation	*nirK* in Clusters I, II, V *nirS* in Cluster I	
Paddy 3		Rhizosphere soil	Gray lowland	Before irrigation	*nirK* in Clusters I, II, V *nirS* in Cluster I	
Paddy 4		Rhizosphere soil	Gray lowland	After irrigation	*nirK* in Clusters I, II, V *nirS* in Cluster I	
Paddy 5	Tokyo (35°44'N, 139°32'E)	Bulk soil	Andosol	After irrigation	*nirK* in Cluster II	(Tago *et al.*, 2011)
Paddy 6	Kumamoto (32°53'N, 130°45'E)	Bulk soil	Andosol	After irrigation	*nirK* in Cluster II	
Cropland 1	Niigata (37°26'N, 138°52'E)	Bulk soil	Gray lowland	No fertilization	*nirK* in Clusters I, II, V *nirS* in Cluster I	(Wei *et al.*, 2014)
Cropland 2		Bulk soil	Gray lowland	Urea-fertilization	*nirK* in Clusters I, II, V *nirS* in Cluster I	
Cropland 3		Bulk soil	Gray lowland	Organic-manure	*nirK* in Clusters I, II, V *nirS* in Cluster I	
Cropland 4	Yamagata (38°14'N, 140°14'E)	Bulk soil	Gray lowland	Urea-fertilization	*nirK* in Cluster II	(Tago *et al.*, 2011)
Cropland 5	Tokyo (35°44'N, 139°32'E)	Bulk soil	Andosol	Urea-fertilization	*nirK* in Cluster II	
Cropland 6	Kumamoto (32°53'N, 130°45'E)	Bulk soil	Andosol	Urea-fertilization	*nirK* in Cluster II	
Forest 1	Hokkaido (43°19'N, 143°30'E)	Bulk soil	Cambisol	—	*nirK* in Clusters I, II, V *nirS* in Cluster I	(Urakawa *et al.*, 2015)
Forest 2	Hokkaido (43°23'N, 144°39'E)	Bulk soil	Andosol	—	*nirK* in Clusters I, II, V *nirS* in Cluster I	
Forest 3	Iwate (40°00'N, 140°56'E)	Bulk soil	Andosol	—	*nirK* in Clusters I, II, V *nirS* in Cluster I	
Forest 4	Gunma (36°32'N, 139°25'E)	Bulk soil	Andosol	—	*nirK* in Clusters I, II, V *nirS* in Cluster I	
Forest 5	Miyazaki (32°22'N, 131°05'E)	Bulk soil	Cambisol	—	*nirK* in Clusters I, II, V *nirS* in Cluster I	
Forest 6	Kagoshima (31°32'N, 130°45'E)	Bulk soil	Regosol	—	*nirK* in Clusters I, II, V *nirS* in Cluster I	
