# Supplementary material for: Distinct Community Composition of Previously Uncharacterized Denitrifying Bacteria and Fungi across Different Land-Use Types
Source: Microbes Environ. 2020 Jan 30;35(1):ME19064. doi: 10.1264/jsme2.ME19064 (PMC7104279; doi:10.1264/jsme2.ME19064)
Supplement: Supplementary file 1 — Supplementary Material [file 35_19064_s1.pdf]

Supplementary Materials for

Distinct Community Composition of Previously Uncharacterized Denitrifying Bacteria and Fungi across Different Land-Use Types

Reiko Fujimura, Yoichi Azegami, Wei Wei, Hiroko Kakuta, Yutaka Shiratori, Nobuhito Ohte, Keishi Senoo, Shigeto Otsuka, and Kazuo Isobe*

*Corresponding author. Email: akisobe@mail.ecc.u-tokyo.ac.jp

This PDF includes:

Tables S1 to S3
Figs. S1 to S3

Table S1. Total number of sequence read.

Sample name	Total count of read/sample			
	nirK in Cluster I	*nirK* in Cluster II	*nirK* in Cluster V	*nirS* in Cluster I
Paddy 1	20,536	107,953	n.d.	223,115
Paddy 2	11,464	96,324	n.d.	235,529
Paddy 3	41,551	141,565	n.d.	304,658
Paddy 4	62,815	153,508	n.d.	319,663
Paddy 5	N.D.	9,546	N.D.	N.D.
Paddy 6	N.D.	7,050	N.D.	N.D.
Cropland 1	217,267	84,171	24,698	89,264
Cropland 2	167,115	71,432	5,325	102,131
Cropland 3	245,341	34,284	2,577	162,711
Cropland 4	N.D.	23,447	N.D.	N.D.
Cropland 5	N.D.	34,264	N.D.	N.D.
Cropland 6	N.D.	5,177	N.D.	N.D.
Forest 1	31,460	105,321	763	13,235
Forest 2	40,205	155,835	1,911	12,199
Forest 3	94,418	126,066	1,854	38,494
Forest 4	11,383	98,303	11,828	260,290
Forest 5	53,146	131,994	6,537	31,239
Forest 6	86	179,698	40,749	339,110

N.D.: not determined

n.d.: not detected.

Table S2. Total number of taxa per 1,850 read.

Sample name	Total number of taxa			
	nirK in Cluster I	*nirK* in Cluster II	*nirK* in Cluster V	*nirS* in Cluster I
Paddy 1	99	474	n.d.	112
Paddy 2	95	396	n.d.	87
Paddy 3	100	226	n.d.	134
Paddy 4	87	255	n.d.	83
Paddy 5	N.D.	516	N.D.	N.D.
Paddy 6	N.D.	552	N.D.	N.D.
Cropland 1	36	374	49	107
Cropland 2	39	272	37	103
Cropland 3	38	146	44	79
Cropland 4	N.D.	750	N.D.	N.D.
Cropland 5	N.D.	736	N.D.	N.D.
Cropland 6	N.D.	466	N.D.	N.D.
Forest 1	99	380	n.d.	78
Forest 2	114	340	51	44
Forest 3	52	107	32	16
Forest 4	79	129	26	16
Forest 5	117	376	46	29
Forest 6	N.D.	244	17	42

N.D.: not determined.

n.d.: not detected.

Table S3. Physicochemical properties of samples.

Sample name	GWC	pH (H_2O)	NO_3^- mg-N kg^{-1}	NH_4-N mg-N kg^{-1}
Paddy 1	0.53*	6.7	n.d.*	2.35*
Paddy 2	0.54*	6.6	n.d.*	n.d.*
Paddy 3	N.D.	N.D.	N.D.	N.D.
Paddy 4	N.D.	N.D.	N.D.	N.D.
Paddy 5	0.46	6.8	0.60	37.6
Paddy 6	0.52	6.8	1.70	39.2
Cropland 1	0.32	6.7	4.33	4.6
Cropland 2	0.30	6.7	19.4	149
Cropland 3	0.32	7.0	11.8	116
Cropland 4	0.14	6.6	3.80	0.77
Cropland 5	0.35	5.7	23.2	4.03
Cropland 6	0.31	5.4	47.3	15.4
Forest 1	0.47	6.0	0.62	2.02
Forest 2	0.54	5.5	1.75	0.91
Forest 3	0.58	4.8	1.48	17.9
Forest 4	0.63	5.0	0.20	14.8
Forest 5	0.54	5.0	0.26	8.30
Forest 6	0.19	5.5	0.13	4.14

* Data from Ito *et al*. (2013) at the same sampling site and season.

N.D.: not determined

n.d.: not detected.

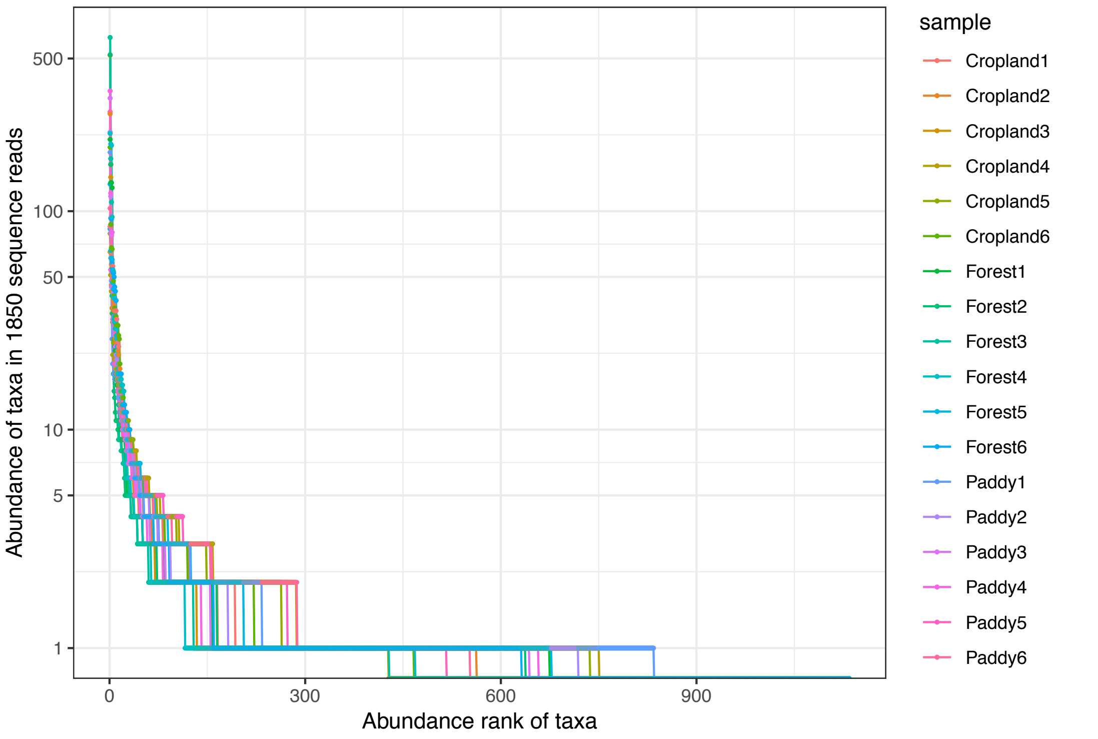

Fig. S1. Rank abundance curve of Cluster II in *nirK* sequences. The y-axis showing the log scale represents the sequence read count of each taxa, and the x-axis represents the rank of the taxa.

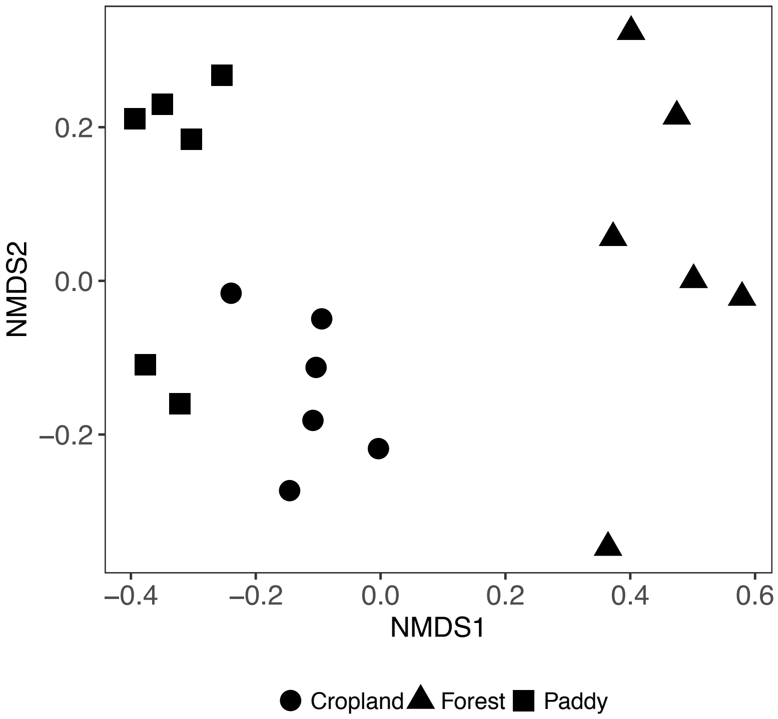

Fig. S2. Nonmetric multidimensional scaling (NMDS) ordination of variation in communities of denitrifying bacteria having *nirK* in Cluster II based on the Bray–Curtis dissimilarity index among different land-use management types. The stress value is 0.13. The legends of the plots are indicated in the figure.

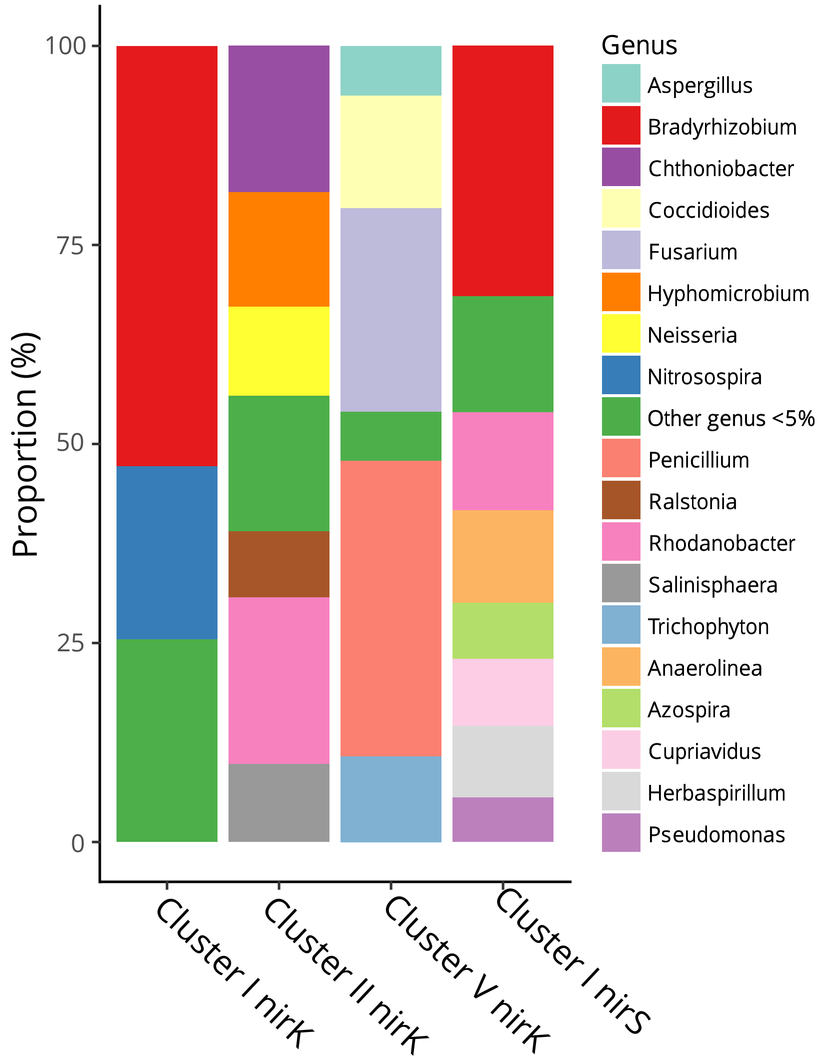

Fig. S3. Assigned genus-level taxonomic composition of denitrifying bacteria and fungi having *nirK* or *nirS* belonging to each cluster among all samples. The y-axis indicates the relative abundances of the taxa. The color legends are shown in the figure and asterisks are added on the fungal taxa names.
